# Predictors of in vitro response to somatostatin and dopamine receptor‐targeted treatments are lacking in clinically non‐functioning pituitary tumors

**DOI:** 10.1111/jne.70272

**Published:** 2026-09-24

**Authors:** Claudia Campana, Jessica Amarù, Diego Criminelli Rossi, Anna Arecco, Paolo Nozza, Mara Boschetti, Gianluigi Zona, Diego Ferone, Marica Arvigo, Federico Gatto

**Affiliations:** ^1^ Endocrinology Unit, Department of Internal Medicine and Medical Specialties (DIMI), School of Medical and Pharmaceutical Sciences University of Genova Genoa Italy; ^2^ Department of Neurosurgery IRCCS AOM – Policlinico San Martino Genoa Italy; ^3^ Pathology Unit IRCCS AOM – Policlinico San Martino Genoa Italy; ^4^ Endocrinology Unit IRCCS AOM – Policlinico San Martino Genoa Italy; ^5^ Neurosurgery Unit, Department of Neuroscience, Ophthalmology, Genetics and Childhood and Maternal sciences (DiNOGMI) University of Genova Genoa Italy

**Keywords:** chimeric molecules, dopamine receptor, medical treatment, non‐functioning pituitary tumor, somatostatin receptor

## Abstract

Clinically non‐functioning pituitary neuroendocrine tumors (NF‐PitNETs) lack effective medical therapies, and the role of somatostatin receptor ligands (SRLs) and dopamine agonists (DAs) in this setting is controversial. We evaluated the in vitro response of NF‐PitNETs to multiple SRL and/or DA treatments, focusing on the role of receptor expression and tumor characteristics in driving tumor response. Forty‐four NF‐PitNET primary cultures were treated (72 h, 10 nM) with octreotide (OCT), pasireotide (PAS), OCT + PAS, BIM‐53097 (D2R agonist), BIM‐53097 + PAS, and BIM‐23B065 (SSTR2/SSTR5/D2R preferential ligand) to evaluate cell proliferation inhibition. Tumors were considered responders to a treatment when a ≥20% reduction in cell proliferation was observed (vs. control); responder tumors to at least one tested condition were classified into the “responder group” for further analysis. Somatostatin (SSTRs) and dopamine type 2 receptor (D2R) expression were evaluated through immunohistochemistry. Data on radiological invasiveness, proliferation indices (Ki67%, mitoses), and p53 immunostaining were collected. Tumor grade was determined using Trouillas and PANOMEN‐3 classifications. Median patient age at surgery was 60.3 years (IQR 49.7–70.7), 29 were males (71%). Overall, no significant in vitro inhibition of cell proliferation was observed [ranging from −4.2% (OCT and PAS) to −7.4% (BIM‐53097 + PAS)]. However, 13 cultures (30%) were included in the “responder group” [mean inhibition ranging from −18.6% (OCT) to −27.7% (BIM‐53097 + PAS)]. D2R was the most expressed receptor (median IRS 8, IQR 6–8), followed by SSTR1 and SSTR2 (median IRS 4, IQR 3–6); SSTR3 and SSTR5 expression was low. In the whole cohort, OCT and PAS efficacy directly correlated with SSTR2 (*p* = .013 and *p* = .027, respectively). D2R and SSTR2 expression was higher in responders vs. non‐responders to BIM‐23B065 (*p* = .017 and *p* = .042, respectively). ROC curve analysis discriminates BIM‐23B065 responders based on D2R and SSTR2 expression with acceptable ability (AUC = 0.784 and AUC = 0.743, respectively). However, the “responder group” did not differ significantly from the “non‐responder group” with respect to patient demographics, tumor clinical–pathological characteristics, receptor expression, and grading. In conclusion, we confirm the limited efficacy of SRL and DA treatment in NF‐PitNETs. D2R and SSTR2 can discriminate tumors responsive to BIM‐23B065. As concerns the other tested compounds, none of the tumor parameters evaluated demonstrated robust predictive value for NF‐PitNET in vitro response.

## 1 INTRODUCTION

Pituitary neuroendocrine tumors (PitNETs) account for 10–15% of intracranial tumors, and about one third of all PitNETs are clinically non‐functioning (NF) tumors.^1^ To date, the only available treatments for NF‐PitNETs are surgical resection and radiotherapy, whereas no pharmacological treatment has been approved so far.^2^ Of note, it has been shown (both at mRNA and protein level) that NF‐PitNETs express high levels of dopamine type 2 receptor (D2R) and somatostatin receptors (SSTRs), in particular SSTR subtype 3 (SSTR3) and subtype 2 (SSTR2).^3^, ^4^, ^5^, ^6^, ^7^ In this light, different medical treatments, approved for other PitNET histotypes and targeting the abovementioned receptors, have been investigated. Dopamine agonists (DAs, such as cabergoline) mainly act on D2R and have been investigated both in vivo and in vitro.^2^ While most data reported in the literature describe hardly any effect of DA treatment in NF‐PitNETs,^8^ a prospective clinical study carried out by Batista and colleagues in 2019 showed a significantly longer time to tumor progression in treated patients compared to observation only.^9^ However, no predictive factors for DA response were identified.^9^ On the other hand, somatostatin receptor ligands (SRLs) may activate one or more SSTR subtypes, depending on the receptor affinity of each molecule.^10^ First‐generation SRLs, octreotide (OCT) and lanreotide (LAN), show high affinity for SSTR2 and moderate affinity for SSTR5, whereas pasireotide (PAS) has high affinity for multiple SSTRs (SSTR5 > SSTR2 > SSTR3).^11^ A number of in vitro and in vivo studies on animal models have suggested a potential role for these molecules, particularly PAS.^12^, ^13^ However, a recent prospective randomized clinical trial did not show any significant difference between LAN therapy and placebo in NF‐PitNET progression (despite a positive imaging for SSTR2 expression), while clinical data from prospective studies are currently lacking for PAS.^14^

Based on this evidence, we aimed to evaluate in a large cohort of NF‐PitNETs the in vitro response to multiple SRL and/or DA treatments and to investigate clinical and pathological factors that could predict treatment response.

## 2 MATERIALS AND METHODS

### 2.1 Reagents

OCT and PAS were obtained from Novartis Pharma A.G. (Basel, Switzerland). The D2R agonist BIM‐53097 and the SSTR2/SSTR5/D2R chimeric compound BIM‐23B065 were kindly provided by Biomeasure Incorporated/IPSEN (Milford, MA). Of note, BIM‐53097 and BIM‐23B065 are molecules developed for research use, currently not approved for clinical practice.

### 2.2 Patients and tumors

Forty‐four NF‐PitNET primary cultures obtained from 41 patients who underwent trans‐sphenoidal surgery at the Neurosurgery Unit of our Institution (IRCCS AOM‐Policlinico San Martino, Genoa, Italy) were included in the study. Three NF‐PitNET primary cultures were obtained from recurrent tumors who underwent a second surgery (patients n.3, n.5, and n.20).

Inclusion criteria of the study were (1) availability of enough viable cells to establish a primary culture and (2) adequate cell number to test in the same experiment, at least in triplicate, the antiproliferative effect of treatment with SRLs, D2R, and SSTR/D2R compounds (alone and in combination).

General characteristics of the patients, as well as histopathological information of the tumors are reported in Table 1.

**TABLE 1:** General characteristics of the patients, tumor characteristics, and receptor expression.

N. tumor	N. patient	Age at surgery	Tumor size^a^	Knosp grade	Invasiveness^b^	Score Ki‐67^c^	Score mitosis^d^	Score p53^e^	Proliferative^f^	Hormone IHC positiviy	D2R IRS	SSTR1 IRS	SSTR2 IRS	SSTR3 IRS	SSTR5 IRS
1	1	41	1	3	1	0	0	0	0	LH/FSH	9	6	8	3	2
2	2	45	1	2	0	0	0	0	0	LH/FSH	12	4	8	0	4
3	3	58	1	4	1	0	0	0	0	ACTH	8	4	6	1	3
4	4	77	1	3	1	0	0	0	0	LH/FSH	9	3	3	2	2
5	5	75	1	1	0	0	0	0	0	LH/FSH	9	6	6	2	2
6	6	45	1	3	1	0	0	0	0	LH/FSH	9	4	8	1	2
7	7	49	0	0	0	0	0	0	0	LH/FSH	4	6	8	0	4
8	8	82	1	1	0	0	0	0	0	ACTH	4	8	6	2	1
9	9	35	1	1	0	0	0	0	0	LH/FSH	6	1	3	4	1
10	10	78	1	4	1	0	0	0	0	LH/FSH	8	3	2	3	1
11	11	55	1	3	1	0	0	0	0	LH/FSH	8	3	4	1	6
12	12	68	1	2	0	0	0	0	0	LH/FSH	8	6	6	4	4
13	13	54	1	2	0	0	0	0	0	LH/FSH	9	6	6	4	6
14	14	61	1	4	1	1	0	0	0	LH/FSH	6	2	3	1	2
15	15	66	1	3	1	0	0	0	0	LH/FSH	9	4	2	3	4
16	16	58	1	2	0	1	0	0	0	LH/FSH	2	9	3	1	8
17	17	67	1	3	1	0	0	0	0	LH/FSH	8	4	3	4	1
18	18	79	1	1	0	0	0	0	0	LH/FSH	6	2	6	1	1
19	19	55	1	3	1	0	0	0	0	LH/FSH	6	3	6	1	2
20	20	59	1	3	1	0	0	0	0	LH/FSH	12	6	12	0	4
21	5^g^	78	1	1	0	0	0	0	0	LH/FSH	6	6	2	1	1
22	21	34	1	1	0	1	1	0	1	LH/FSH	8	3	3	2	4
23	22	60	1	3	1	1	0	1	1	LH/FSH	4	2	6	1	1
24	23	79	1	1	0	0	0	0	0	LH/FSH	4	12	4	2	6
25	24	76	1	2	0	0	0	1	0	LH/FSH	6	8	2	2	3
26	25	44	1	2	0	0	0	0	0	None	8	4	4	2	8
27	26	50	1	1	0	0	0	0	0	LH/FSH	6	4	1	2	1
28	3^g^	63	1	4	1	0	0	0	0	ACTH	8	6	4	0	4
29	27	77	1	2	0	0	0	0	0	LH/FSH	9	4	2	1	2
30	28	48	1	2	0	0	0	0	0	LH/FSH	8	6	6	2	3
31	29	58	1	2	0	0	0	0	0	LH/FSH	8	2	8	1	2
32	30	55	1	1	0	1	0	1	1	LH/FSH	4	4	6	2	2
33	31	62	1	1	0	1	0	1	1	LH/FSH	8	6	2	3	0
34	32	68	1	3	1	0	0	0	0	LH/FSH	9	4	6	1	2
35	33	71	1	3	1	1	1	0	1	LH/FSH	4	2	4	1	3
36	34	65	1	4	1	0	0	0	0	LH/FSH	4	4	3	2	2
37	35	42	1	3	1	0	0	0	0	None	6	2	4	0	8
38	36	79	1	4	1	0	0	0	0	LH/FSH	6	6	4	2	6
39	37	75	1	2	0	0	0	0	0	LH/FSH	8	9	3	2	2
40	38	63	1	4	1	0	0	0	0	FSH	6	2	8	2	0
41	39	53	1	3	1	0	0	0	0	LH/FSH	6	8	4	3	4
42	40	50	1	4	1	0	0	0	0	FSH	3	12	9	0	3
43	41	60	1	1	0	0	0	0	0	FSH	n.a.	n.a.	n.a.	n.a.	n.a.
44	20^g^	64	1	4	1	0	0	0	0	LH/FSH	n.a.	n.a.	n.a.	n.a.	n.a.

Abbreviations: D2R, dopamine receptor subtype 2; IRS, immunoreactivity score; SSTR1‐5, somatostatin receptor subtype 1–5.

^a^ 0: tumor diameter <10 mm, 1: tumor diameter ≥10 mm.

^b^ 0: non‐invasive tumor, 1: invasive tumor.

^c^ 0: Ki‐67 <3%, 1: Ki‐67 ≥3%.

^d^ 0: mitosis ≤2/10 high power field (HPF), 1: mitosis >2 HPF.

^e^ 0: negative p53 (≤10 strongly positive nuclei/10 HPF), 1: positive p53 (>10 strongly positive nuclei/10 HPF).

^f^ 1: presence of at least two between Ki‐67, mitosis or p53 criteria, as previously defined.

^g^ Sample obtained from a recurrent tumor, the patient number has been attributed at the first surgery.

Briefly, the majority of patients were males (70.7%) and the median age at time of surgery was 60.3 years (IQR 49.7–70.7 years). All tumors were assessed for size (micro vs. macro), radiological invasiveness (Knosp grade), proliferation indices (Ki67%, mitoses), and p53 immunostaining. All patients, except patient n.7, had a macroadenoma and 22 lesions (50%) were invasive (Knosp grade 3 or 4). Only five tumors exhibited high proliferative activity according to the classification of Trouillas and colleagues (two out of three parameters among: Ki67 ≥3%, mitosis >2/10 HPF, p53 >10/10 HPF).^15^ Based on this data, NF‐PitNETs were categorized according to Trouillas' classification. Only tumors n.23 and n.35 showed invasive and proliferative lesions (grade 2b; Table 1).

Moreover, all cases were individually assessed and scored according to the PANOMEN‐3 grade at the first outpatient visit after surgery, as previously described.^16^ In detail, three patients (7%) were grade 0, five patients (11%) were grade 1, 22 patients (50%) were grade 2, and 14 patients (32%) were grade 3. Of note, two out of three cases in which two surgeries were performed were grade 2 both at the first and second surgery, whereas one was grade 3 at the first surgery and grade 2 at the second intervention.

Of note, 12 patients experienced tumor regrowth during follow‐up. Of these, five patients underwent a second surgery, five patients underwent radiotherapy (RT), while in two cases active follow‐up was the approach. In five patients, the DA cabergoline was administered after the first surgery; in four out of five patients the tumor showed regrowth during DA treatment (median dose 1 mg/week, IQR 0.25–1.00).

As concerns pituitary function after first surgery, the majority of patients had at least one pituitary deficit (*n* = 30, 68%). In detail, 21 patients had central hypogonadism, 16 patients had central hypothyroidism, and 13 patients had central hypocortisolism (overall, 16 patients had more than one pituitary hormone deficiency).

In all tissues (*n* = 44), immunoreactivity (IR) for anterior pituitary hormones was evaluated. The vast majority showed positive staining for both LH and FSH (*n* = 36, 81.8%), three tumors only for FSH (samples n.40, n.42, and n.43), cases n.26 and n.37 showed no immunohistochemistry (IHC) positivity for anterior pituitary hormones, while three cases showed positive ACTH staining (tumors n.3, n.8, and n.28) and were classified as silent ACTH‐secreting PitNETs (Table 1).

The study was conducted in accordance with the recommendations of the Declaration of Helsinki, and all patients gave written informed consent to use clinical data for research purposes. This study was approved by the local ethical committee (Comitato Etico Territoriale Regione Liguria, CET‐Liguria register number: 360/2019).

### 2.3 Immunohistochemistry

SSTR and D2R expression was evaluated in 42 out of 44 tumor samples, depending on the availability of formalin‐fixed paraffin‐embedded (FFPE) tissue, using the Dako EnVision® + Dual Link System‐HRP (DAB+) kit (Dako/Agilent, Santa Clara, CA), as previously reported.^17^ To detect SSTR2 and SSTR5, 1:200 dilution of rabbit anti‐SSTR2 (RRID: AB_2737601) and rabbit anti‐SSTR5 (RRID: AB_10859946) monoclonal antibodies (UMB‐1 and UMB‐4, respectively, Abcam, Cambridge, UK) were used. To detect SSTR1 and SSTR3, the rabbit anti‐SSTR1 (RRID: AB_2196035) and the rabbit anti‐SSTR3 (RRID: AB_778014) polyclonal antibodies (both from Abcam, Cambridge, UK) were used at 1:50 and 1:1000 dilutions, respectively. To detect D2R, 1:150 dilution of the mouse anti‐D2R monoclonal antibody (RRID: AB_668816, Santa Cruz Biotechnology, Inc., Santa Cruz, CA) was used. Slides were counter‐stained with hematoxylin. Receptor staining on tumor samples was assessed using a semi‐quantitative immunoreactivity scoring system (IRS), as previously described.^17^

### 2.4 NF‐PitNET primary cultures

Primary cultures from NF‐PitNETs were set according to a protocol developed in our laboratory.^18^ To assess the purity of our primary cultures and to further validate our method, 21 out of 44 NF‐PitNET samples included in the study (in which enough material was available) were analyzed by flow cytometry immediately after cell dispersion, using chromogranin A (CgA) as marker of neuroendocrine cells, and CD45 as marker of hematopoietic cells. In detail, 7.5 × 10^5^ to 1 × 10^6^ cells (depending on total cell yield) were stained with mouse AlexaFluor647‐conjugated anti‐chromogranin A (LK2H10) monoclonal antibody (RRID: AB_3290922, Biotechne/Novus Biologicals, Minneapolis, MN) and mouse Fluorescein isothiocyanate (FITC)‐conjugated anti‐CD45 antibody (RRID: AB_10645157, Beckman Coulter, Brea, CA). The flow cytometry analysis of NF‐PitNET primary cultures showed 90.3% CgA‐positive cells (interquartile range: 77.1%–96.7%) and 6.6% of CD45‐positive cells (interquartile range: 2.1%–20.9%), demonstrating the high purity of the cell cultures.

### 2.5 Cell proliferation

NF‐PitNET cells (3 × 10^4^ cells/well) were seeded in 96‐well plates and incubated at 37°C in a humidified 5% CO_2_ atmosphere for 24 h. Cells were treated in triplicate with OCT, PAS, BIM‐53097, BIM‐23B065, at the concentration 10 nM, alone or in equimolar combination, for 72 h. We used the 10 nM concentration because it represents the concentration most commonly employed in human pituitary tumor cultures. This concentration has been extensively adopted in previous studies evaluating the antisecretory and antiproliferative effects of SRLs and DAs in vitro. It provides near‐maximal receptor activation, allowing direct comparison with the existing literature.^19^, ^20^ Twenty‐four hours before the conclusion of the 72 h incubation, BrdU was added to the cells to evaluate cell proliferation (BrdU Cell Proliferation Assay Kit, Cell Signaling Technology, Danvers, MA) according to the manufacturer's instructions. Primary cultures were considered as responders when an inhibition ≥20% of cell proliferation compared to untreated cells was observed. Moreover, primary cultures with response to at least one tested condition were classified as “responder group” and sub‐analyses were carried out in this subgroup.

### 2.6 Statistical analysis

Results are expressed as median ± interquartile range (IQR) or mean ± standard error (SEM), as appropriate. Statistical analysis was performed using R 4.3.3 software; figures were drawn using GraphPad 9.0.0 (GraphPad Software Inc., San Diego, CA). Mann–Whitney *U* test and Kruskal–Wallis test followed by Dunn's post hoc test were used to analyze differences between groups, as appropriate. Correlation coefficients were calculated using Spearman's rho. Univariate linear regression analysis was performed on data showing significant correlations. The ability of receptor expression to identify responders to the different compounds was evaluated with the Receiver Operating Characteristic (ROC) curve and the related area under the curve (AUC). The best‐fitting cut‐offs were computed using the Youden index. Statistical significance was established at *p* < .05; when multiple testing was performed *p* values were adjusted using the Bonferroni correction.

## 3 RESULTS

### 3.1 D2R and SSTR expression in NF‐PitNET samples

**FIGURE 1:**
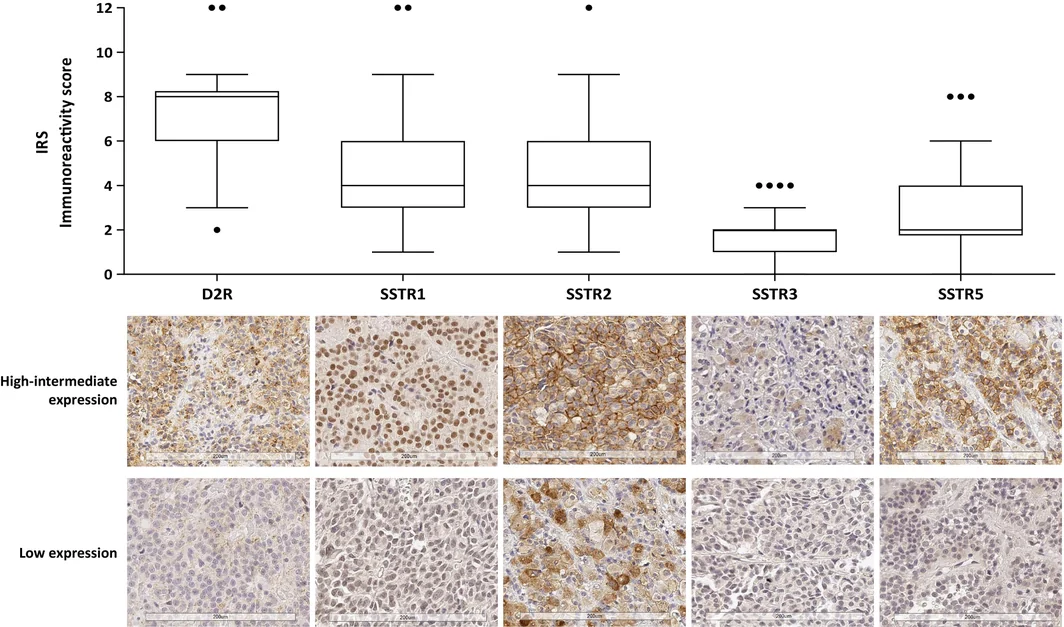
Immunohistochemical evaluation of D2R and SSTR1‐5 in NF‐PitNETs. In the upper part, the Tukey box‐and‐whisker plot shows the distribution of D2R and SSTR1‐5 IRS in the 42 NF‐PitNET samples analyzed. In the lower part, representative images of high‐intermediate (above) and low (below) expression of each D2R or SSTR1‐5 expression in different NF‐PitNETs. Scale bars: 200 μm, magnification: 20×.

IHC evaluation showed a heterogeneous pattern of D2R and SSTR expression in the 42 NF‐PitNET samples analyzed (Figure 1). All samples showed D2R staining, with a high‐intermediate IRS (median 8, IQR 6–8). As expected, the receptor was both on the membrane and in the cytoplasm with a granular staining pattern. All samples showed SSTR1 and SSTR2 positivity, with an intermediate IRS (median IRS = 4, IQR 3–6 for both receptors). SSTR3 was positive in 36 tumors (85.7% of samples) and exhibited a low IRS (median = 2, IQR 1–2). SSTR5 was positive in the vast majority of samples (*n* = 40, 95.2%), showing a low score (median IRS = 2, IQR 2–4) (Figure 1 and Table 1). Staining localization was mainly nuclear for SSTR1, membranous for SSTR2 and SSTR5, whereas SSTR3 showed a prevalent cytoplasmic localization (Figure 1).

We observed a significant negative correlation between SSTR2 and SSTR3 expression (rho = −0.367, *p* = .017), and a significant positive correlation between SSTR1 and SSTR5 IRS (rho = 0.308, *p* = .047).

### 3.2 Effect of SRLs, D2R, and SSTR/D2R experimental compounds on cell proliferation

The responses of the primary cultures to the experimental compounds, tested at a single concentration of 10 nM, were highly heterogeneous.

**FIGURE 2:**
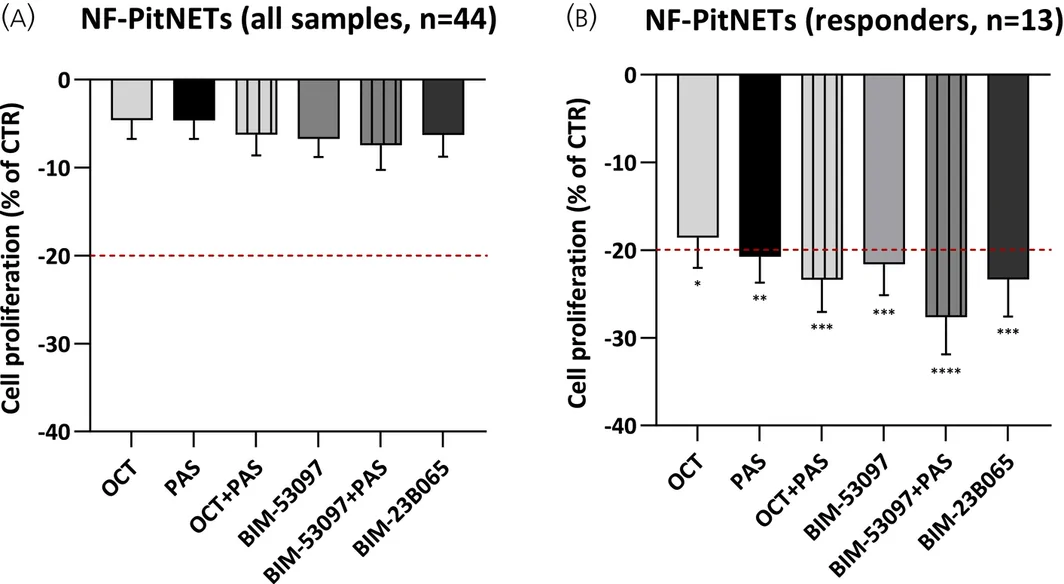
In vitro effect of all tested compound (10 nM), alone or in combination, on cell proliferation in 44 primary cultures (panel A) and in the “responder group” (*n* = 13; panel B) from NF‐PitNETs. Data are expressed as mean ± SEM and reported as percentage inhibition of control cells. The red line indicates the threshold corresponding to a 20% reduction in cell proliferation relative to untreated cells. CTR, control; OCT, octreotide; PAS, pasireotide; BIM‐53097, D2R agonist; BIM‐23B065, SSTR2/SSTR5/D2R chimeric compound; *p < .05, **p < .01, ***p < .001, ****p < .0001 vs. CTR.

Overall, considering the mean results on cell proliferation of all 44 NF‐PitNET cultures, no tested compound, alone or in combination, was able to significantly inhibit cell proliferation, ranging from −4.6% (OCT and PAS alone) to −7.4% (BIM‐23097 + PAS) (Figure 2A). Of note, the effect induced by each tested compound was directly correlated with the effect induced by all other treatments (Spearman's rho ranging 0.612–0.846, *p* < .0001; Figure S1, Supporting Information).

Thirteen NF‐PitNET cultures (29.5%) were assigned to the “responder‐group,” as defined in Section 2. In this group, all compounds induced a statistically significant inhibition of cell proliferation compared to untreated cells (OCT: −18.6% ± 3.5, *p* < .05; PAS: −20.8% ± 3.0, *p* < .01; OCT + PAS: −23.4% ± 3.7, BIM‐53097: −21.6% ± 3.5, BIM‐23B065: −23.4% ± 4.2, all *p* < .001; BIM‐53097 + PAS: −27.7% ± 4.2, *p* < .0001) (Figure 2B). In detail, 6 out of 13 cultures were considered responders to OCT, 7 to PAS, 7 to OCT + PAS, 9 cultures responded to BIM‐53097, 10 to BIM‐53097 + PAS, and 8 to BIM‐23B065 (see Table S2).

Of note, five patients were treated in vivo with DA after first surgery. One out of the four DA treated patients that experienced tumor regrowth in vivo showed a significant response to BIM53097 in vitro. On the other hand, the only patient (out of five) who did not show recurrence after DA treatment did not show any significant response in vitro.

### 3.3 In vitro response and receptor expression

#### 3.3.1 NF‐PitNET whole cohort

**FIGURE 3:**
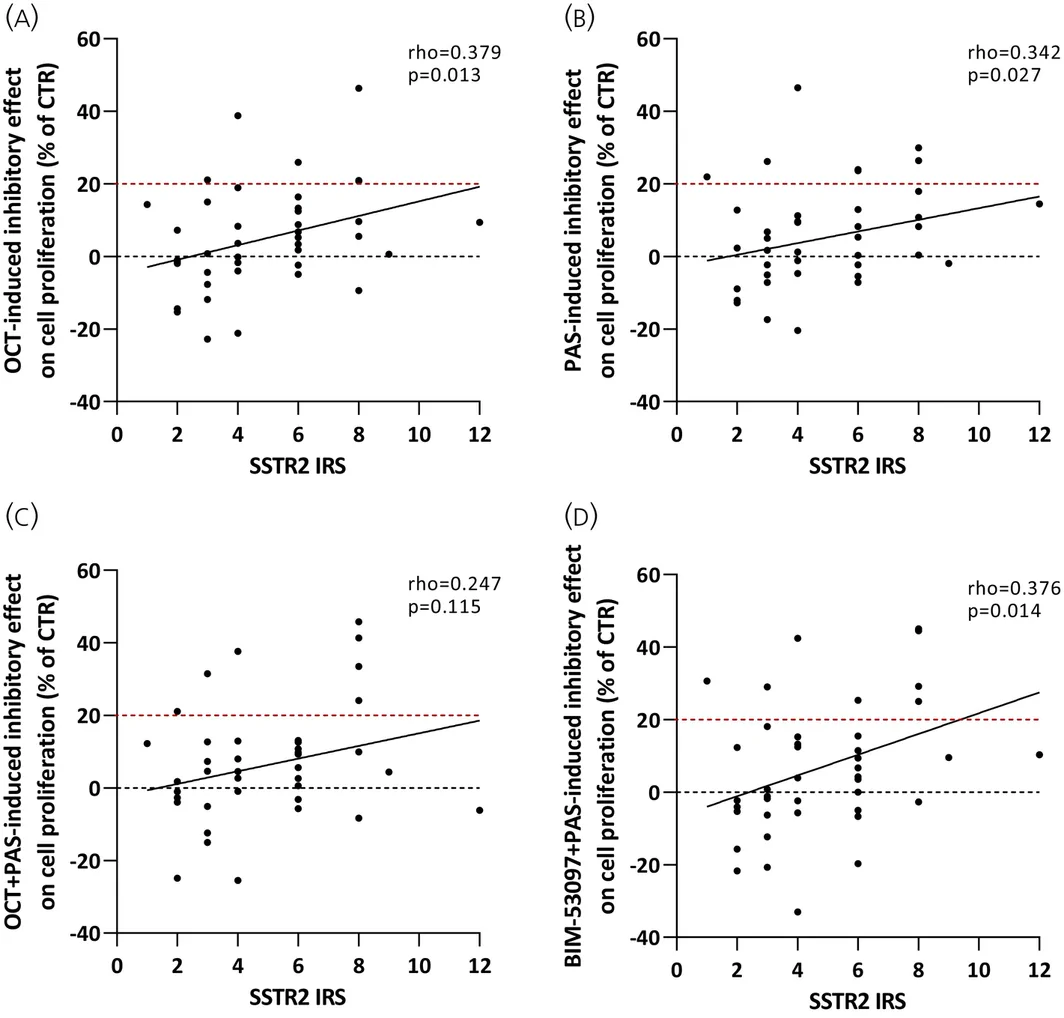
Correlation between inhibitory effect of OCT (panel A), PAS (panel B), OCT + PAS (panel C) and BIM‐53097 + PAS (panel D) and SSTR2 expression in 42 primary cultures from NF‐PitNETs. The red line indicates the threshold corresponding to a 20% reduction in cell proliferation relative to untreated cells. CTR, control; OCT, octreotide; PAS, pasireotide; BIM‐53097, D2R agonist; IRS, immunoreactivity score. In panels A and B, two data points are nearly overlapping on both axes. Similarly, in panel D this happens for three data points. Therefore, the number of visible black dots does not correspond to the number of data points described in the text.

Considering the 42 NF‐PitNET cultures in which receptor expression was available, the effect of OCT and PAS monotherapy was positively correlated with SSTR2 expression (OCT: rho = 0.379, *p* = .013; PAS: rho = 0.342, *p* = .027; Figure 3), while no statistically significant correlation was observed between SSTR2 and OCT + PAS (rho = 0.247, *p* = .115; Figure 3C). At linear regression analysis, SSTR2 was positively associated with the response to OCT (*β* = 2.01, 95% CI 0.23–3.79; *p* = .028), explaining 12% of the observed variation (Table S1).

Interestingly, SSTR2 expression was not significantly different in patients classified as responders to OCT and PAS (both alone and in combination) vs. the non‐responders, while D2R expression was significantly higher in responders to OCT + PAS compared to non‐responders (median 9 [IQR 7–9] vs. 6 [IQR 5–8], *p* = .050; Table 2). At ROC curve analysis, neither SSTR2 nor D2R expression was able to differentiate responders to OCT and PAS alone from non‐responders (Figure S2). D2R expression differentiated responders from non‐responders to OCT + PAS with mild‐acceptable ability, although with low sensitivity (AUC = 0.733, 95% CI 0.527–0.939; cut‐off IRS 8.5: sensitivity 57%, specificity 83%; Figure S2).

**TABLE 2:** Correlation analysis and group comparison between the effect of all tested compounds (10 nM), alone or in combination, on cell proliferation and receptor expression, patient and tumor characteristics, Trouillas and PANOMEN‐3 classifications. (A) Correlation analysis in the whole cohort and in the responder‐group. (B) Comparison between tumor responders vs. non‐responders to the different treatments.

(A) Correlation analysis		Receptor expression					Patient and tumor characteristics						
		D2R IRS	SSTR1 IRS	SSTR2 IRS	SSTR3 IRS	SSTR5 IRS	Age at surgery	Knosp	Score Ki‐67^a^	Score mitosis^b^	Score p53^c^		
Whole cohort (*n* = 44)	OCT	‐	‐	rho = 0.379 *p* = .013	‐	‐	‐	‐	‐	‐	‐		
	PAS	‐	‐	rho = 0.342 *p* = .027	‐	‐	rho = −0.393 *p* = .009	‐	‐	‐	‐		
	OCT + PAS	‐	‐	‐	‐	‐	rho = −0.422 *p* = .005	‐	‐	‐	‐		
	BIM‐53097	‐	‐	‐	rho = −0.310 *p* = 0.046	‐	rho = −0.363 *p* = 0.016	‐	‐	‐	‐		
	BIM‐53097 + PAS	‐	‐	rho = 0.376 *p* = 0.014	‐	‐	rho = −0.321 *p* = 0.034	‐	‐	‐	‐		
	BIM‐23B065	‐	‐	‐	‐	‐	‐	‐	‐	‐	‐		
Responder‐group (*n* = 13)	OCT	‐	rho = −0.585 *p* = .045	‐	‐	‐	‐	‐	‐	‐	‐		
	PAS	‐	rho = −0.770 *p* = .003	‐	‐	‐	‐	‐	‐	‐	‐		
	OCT + PAS	‐	‐	‐	‐	‐	‐	‐	‐	‐	‐		
	BIM‐53097	‐	‐	‐	‐	‐	‐	‐	rho = −0.597 *p* = .031	‐	‐		
	BIM‐53097 + PAS	‐	‐	‐	‐	‐	‐	‐	rho = −0.751 *p* = .003	‐	‐		
	BIM‐23B065	rho = 0.608 *p* = 0.036	‐	‐	‐	‐	‐	‐	rho = −0.646 *p* = .017	‐	‐		

(B) Group comparison		Receptor expression					Patient and tumor characteristics						
		D2R IRS	SSTR1 IRS	SSTR2 IRS	SSTR3 IRS	SSTR5 IRS	Age at surgery	Knosp	Score Ki‐67^a^	Score mitosi^b^	Score p53^c^	Trouillas	PANOMEN‐3
Responders vs non‐responders to treatments (*n* = 44)	OCT	‐	‐	‐	‐	‐	‐	‐	‐	‐	‐	‐	‐
	PAS	‐	‐	‐	‐	‐	‐	‐	‐	‐	‐	‐	‐
	OCT + PAS	9 vs. 6 *p* = .050	‐	‐	‐	‐	‐	‐	‐	‐	‐	‐	‐
	BIM‐53097	‐	‐	‐	‐	‐	49.5 vs. 62.7 *p* = .032	‐	‐	‐	‐	‐	‐
	BIM‐53097 + PAS	‐	‐	‐	‐	‐	‐	‐	‐	‐	‐	‐	‐
	BIM‐23B065	9 vs. 6 *p* = .017	‐	8 vs. 4 *p* = .042	‐	‐	‐	‐	‐	‐	‐	‐	‐

Abbreviations: D2R, dopamine receptor subtype 2; IRS, immunoreactivity score; SSTR1‐5, somatostatin receptor subtype 1–5.

^a^ 0: Ki‐67 <3%, 1: Ki‐67 ≥3%.

^b^ 0: mitosis ≤2/10 high power field (HPF), 1: mitosis >2 HPF.

^c^ 0: negative p53 (≤10 strongly positive nuclei/10 HPF), 1: positive p53 (>10 strongly positive nuclei/10 HPF).

Regarding the D2R agonist BIM‐53097, we observed a significant negative correlation between its antiproliferative effect and SSTR3 expression (rho = −0.310, *p* = .046). Surprisingly, we did not observe any correlation between the effect of BIM‐53097 and D2R. The combination of BIM‐53097 + PAS positively correlated with SSTR2 (rho = 0.376, *p* = .014; Figure 3D), but not with D2R. At linear regression analysis, SSTR2 expression positively associated with the response to BIM‐53097 + PAS (*β* = 2.86, 95% CI 0.56–5.17; *p* = .016), explaining 14% of the observed variation (Table S1). When we stratified tumors into responders and non‐responders to BIM‐53097 + PAS, SSTR2 did not discriminate the two groups at ROC curve analysis (Figure S2).

**FIGURE 4:**
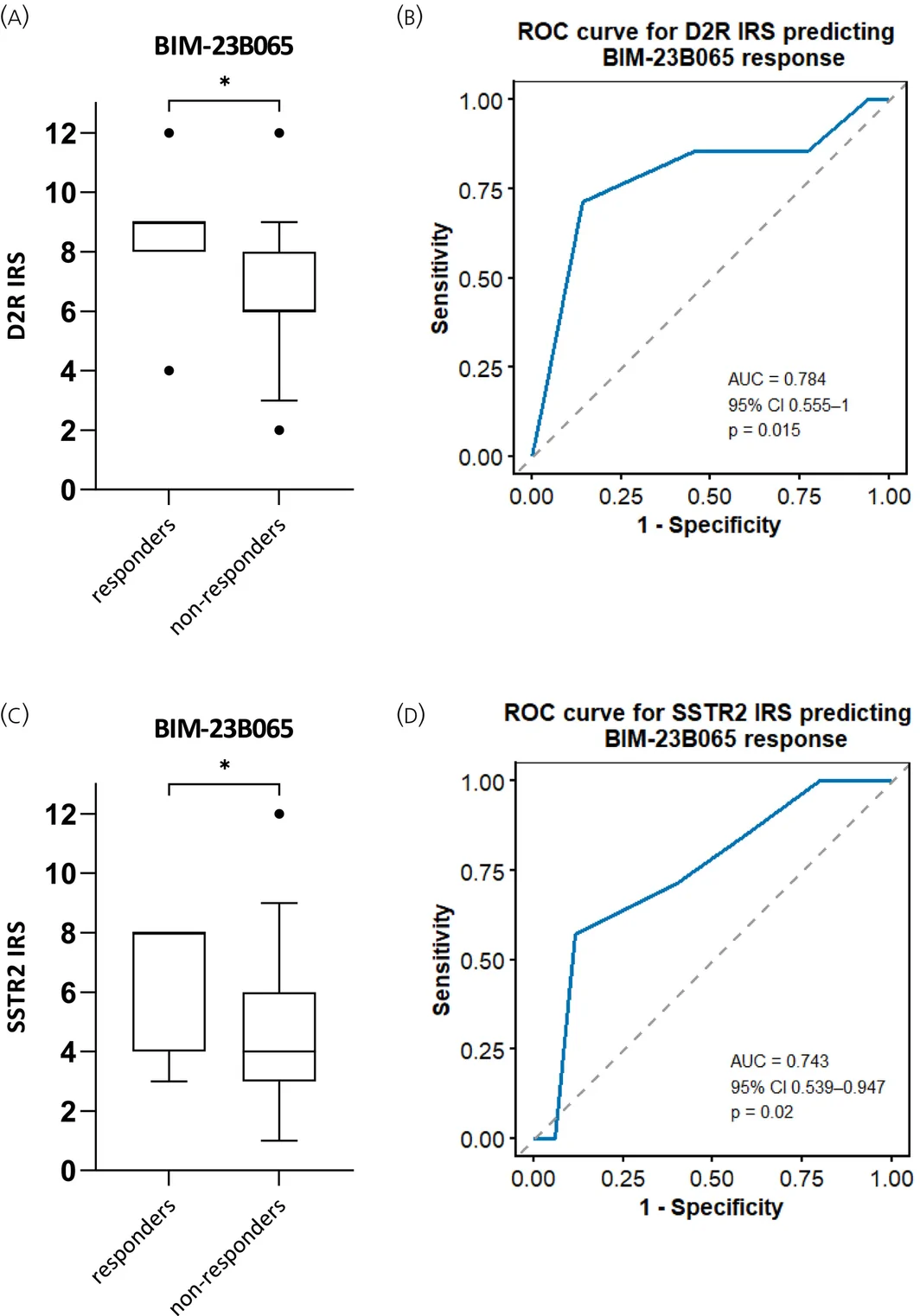
D2R expression in BIM‐23B065 responsive and non‐responsive NF‐PitNETs (panel A) and ROC curve for D2R IRS predicting BIM‐23B065 response (panel B). SSTR2 expression in BIM‐23B065 responsive and non‐responsive NF‐PitNETs (panel C) and ROC curve for SSTR2 IRS predicting BIM‐23B065 response (panel D). CTR, control; BIM‐23B065, SSTR2/SSTR5/D2R chimeric compound; D2R, dopamine receptor type 2; SSTR2, somatostatin receptor subtype 2; IRS, immunoreactivity score; *p < .05.

As concerns the chimeric compound, no significant correlation was observed between the effect of BIM‐23B065 and the expression of D2R and SSTR2. However, both D2R and SSTR2 expression were significantly higher in patients classified as responders to BIM‐23B065 compared to non‐responders (D2R: 9 [IQR 8.5–9] vs. 6 [IQR 6–8], *p* = .017; SSTR2: 8 [IQR 5–8] vs. 4 [IQR 3–6], *p* = .042) (Table 2 and Figure 4). At ROC curve analysis, D2R and SSTR2 expression allowed to differentiate responders from non‐responders to BIM‐23B065 with acceptable ability (D2R: AUC = 0.784, 95% CI 0.5549–1; SSTR2: AUC 0.743, 95%CI 0.5388–0.9469; Figure 4). In detail, setting the cut‐off of D2R IRS to 8.5 allowed to identify responders with 71% sensitivity and 86% specificity, whereas setting the cut‐off of SSTR2 IRS to 7 allowed to identify responders with low sensitivity (56%) but good specificity (89%).

Finally, no correlations were found between the efficacy of all tested compounds and SSTR1 and SSTR5 expression.

#### 3.3.2 “Responder group” (subgroup analysis)

**FIGURE 5:**
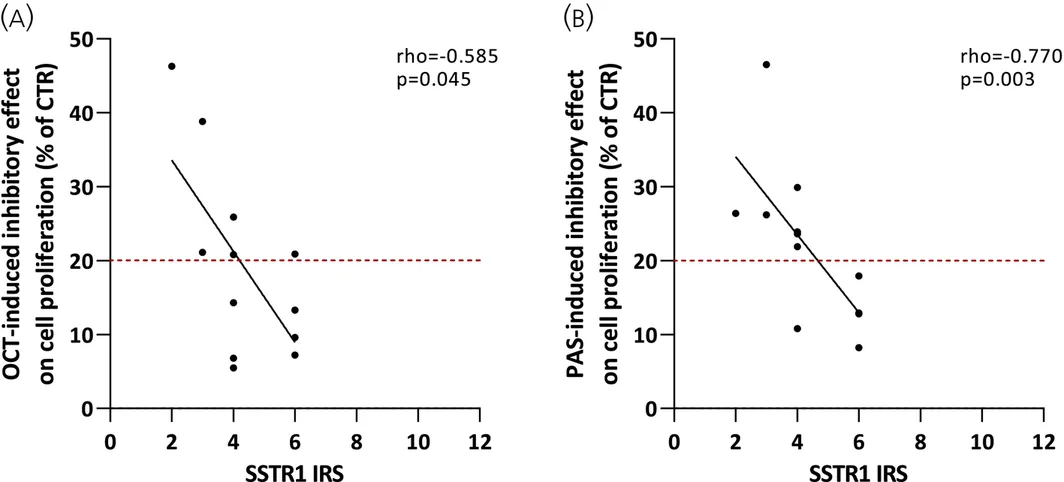
Correlation between inhibitory effect of OCT (panel A) or PAS (panel B) and SSTR1 expression in 13 NF‐PitNET primary cultures, classified as “responder group.” The red line indicates the threshold corresponding to a 20% reduction in cell proliferation relative to untreated cells. CTR, control; OCT, octreotide; PAS, pasireotide; SSTR1, somatostatin receptor subtype 1; IRS, immunoreactivity score. In panel B, two data points are nearly overlapping on both axes; therefore, the number of visible black dots does not correspond to the number of data points described in the text.

Considering only the 13 NF‐PitNET cultures of the “responder group,” the inhibitory effect of OCT and PAS monotherapy negatively correlated with SSTR1 expression (OCT: rho = −0.585, *p* = .045; PAS: rho = −0.770, *p* = .003; Figure 5). As expected, we observed a statistically significant positive correlation between the efficacy of the chimeric compound BIM‐23B065 and D2R expression (rho = 0.608, *p* = .036).

No correlation was observed between the antiproliferative effect of all compounds and SSTR2, SSTR3, and SSTR5 expression.

### 3.4 In vitro response and patients and tumor characteristics

#### 3.4.1 NF‐PitNET whole cohort

Concerning patient demographics, age at time of surgery showed a significant correlation with the in vitro response to the tested treatments. Particularly, primary cultures from elderly patients showed a lower inhibition of cell proliferation to multiple compounds (PAS: rho = −0.393, *p* = .009, OCT + PAS: rho = −0.422, *p* = .005, BIM‐53097: rho = −0.363, *p* = .016, BIM‐53097 + PAS: rho = −0.321, *p* = .034; Table 2). Age at time of surgery was significantly lower in patients classified as responders to BIM‐53097 compared to non‐responders (49.5 vs. 62.7, *p* = .032) but did not reach statistical significance for the other compounds (Table 2).

Regarding patients' sex, no significant differences were observed between females and males in the response to different treatments (data not shown).

No association was observed between response to the tested compounds and Knosp grade or proliferation indices (Table 2). Similarly, treatment response was not significantly different among NF‐PitNET groups based on the Trouillas and PANOMEN‐3 classification (range *p* = .281–.975).

#### 3.4.2 “Responder group” (subgroup analysis)

No significant difference of the in vitro response to the tested treatments was observed depending on gender or age at time of surgery. Of note, a negative correlation was found between age at time of surgery and SSTR2 expression (rho = −0592, *p* = .043).

Interestingly, in this subgroup, invasive tumors showed a numerically higher inhibition of cell proliferation after treatment compared to non‐invasive tumors. This difference reached statistical significance for OCT + PAS (−32.1% vs. −15.8% compared to untreated cells, *p* = .022) and BIM‐53097 treatments (−30.1% vs. −14.3% compared to untreated cells, *p* = .014).

As concerns proliferation indices, a negative correlation was found between Ki‐67 expression and the inhibitory effect on cell proliferation of BIM‐53097 (rho = −0.597, *p* = .031), BIM‐53097 + PAS (rho = −0.751, *p* = .003), and BIM‐23B065 (rho = −0.646, *p* = .017), whereas mitoses and p53 immunostaining did not correlate with the efficacy of any tested compound.

No significant differences in treatment response were observed among NF‐PitNET groups based on the Trouillas and PANOMEN‐3 classifications.

## 4 DISCUSSION

We report a large series of well‐characterized NF‐PitNETs treated in vitro with multiple compounds targeting SSTRs and/or D2R. To our knowledge, this is the largest series of NF‐PitNET primary cultures in which tumor response was assessed testing simultaneously six different treatment conditions, including OCT + PAS and the combination of a D2R agonist (BIM‐53097) + PAS. Despite being an experimental compound not approved for clinical use, the preferential binding affinity of BIM‐53097 resembles that of the clinically available DA cabergoline.

Our NF‐PitNET cohort has been well characterized for SSTR and D2R receptor expression at protein level by use of IHC. In line with previous studies, D2R was the most expressed receptor, showing positivity in all tumor samples and a high‐intermediate expression.^7^, ^9^, ^21^ As concerns SSTRs, SSTR2 and SSTR1 were the most frequently expressed receptors in our cohort, showing an intermediate expression.^5^, ^22^ Surprisingly, SSTR3 had low expression with cytoplasmic localization, although it was expressed in the majority of tumors. This finding is in contrast with previously reported data showing SSTR3 as the predominant SSTR receptor observed in NF‐PitNETs.^4^, ^5^, ^23^, ^24^, ^25^, ^26^, ^27^ Previous studies evaluating SSTR3 protein expression used both polyclonal and monoclonal antibodies, including the monoclonal antibody UMB‐5.^4^, ^5^, ^23^, ^24^, ^25^ Importantly, while monoclonal antibodies for other SSTR subtypes, especially SSTR2, are highly specific, widely used, and lead to reproducible results, the evaluation of SSTR3 using the monoclonal antibody UMB‐5 showed limited specificity, possibly leading to an overestimation of the receptor expression.^28^ As a result, the UMB‐5 monoclonal antibody has been discontinued.^29^ Therefore, after testing the UMB‐5 antibody, we decided to use a polyclonal antibody previously reported to show a specific immunohistochemical signal.^30^ Finally, consistent with previous data, SSTR5 expression in our cohort was low, although it was present in a high percentage of tumors.^5^, ^31^

In our whole cohort, no tested compound showed a significant inhibition of cell proliferation; this is in line with the generally reported lack of efficacy of clinically available SRLs (i.e., octreotide and pasireotide), and the DA cabergoline.

However, we observed that a subgroup of tumors (about one third) showed a significant response to at least one tested condition. Unfortunately, no single predictive factor, among those tested in our study, was able to discriminate with good ability this subgroup of tumors compared to non‐responders. Nevertheless, we identified some associations between the investigated variables and the response to single agent therapy (summarized in Table 2). However, due to the limited number of patients in this group, these results need to be considered as exploratory and to be confirmed by further studies.

Consistent with previous studies, only a small percentage of tumors showed a significant response to OCT and PAS, with a similar response rate between the two compounds.^27^ The efficacy of OCT and PAS was directly correlated to SSTR2, whereas no correlation was observed with other SSTR subtypes, similarly to what was observed in GH‐secreting PitNETs.^17^, ^18^, ^32^, ^33^, ^34^ Again, similarly to previous findings from GH‐secreting PitNETs, the combination of OCT and PAS was not superior to either agent alone.^18^ Anyhow, despite the observed correlations with SSTR2, the expression of this receptor was not a robust predictor to discriminate responder vs. non‐responder tumors to these compounds.

Interestingly, a recent study carried out in a rat model harboring MENX‐associated gonadotroph tumors showed a more pronounced effect of PAS compared to OCT, particularly in female animals.^12^ This sex‐related difference was not observed in our cohort, possibly due to the different model studied (rats vs. humans) and the peculiar tumor type of the abovementioned study (i.e., MENX‐associated tumors).

As expected, a subgroup of NF‐PitNETs showed a statistically significant in vitro response to DA treatment (nine primary cultures). Despite some studies reporting higher D2R expression in tumors showing a satisfactory response to DA, the vast majority of published in vitro and in vivo studies, as well as the present data, did not identify D2R expression as a reliable predictor of response to DA in NF‐PitNETs.^9^, ^35^, ^36^

Similarly to an early study from Florio and colleagues (although conducted testing the BIM‐23A760 molecule), we observed that the efficacy of the chimeric compound was similar to the DA alone.^37^ In this context, D2R and SSTR2 showed a potential role in identifying tumors responder to the chimeric compound BIM‐23B065; the expression of both receptors identified tumors responsive to this compound with high specificity (86% and 89%, respectively), but with moderate‐to‐low sensitivity (71% and 56%, respectively). To our knowledge, no predictive factors of the response to this compound have been previously reported in NF‐PitNETs.

Of note, in the present manuscript we did not evaluate the D2R isoforms. However, these isoforms did not seem to have a crucial role in determining the response to DA agonist or the chimeric compound in NF‐PitNETs.^35^, ^38^, ^39^, ^40^ Furthermore, in our study, we observed a negative correlation between DA agonist response and SSTR3 protein expression. This is a novel finding, which could be driven by the unexpectedly low SSTR3 expression observed in our cohort. Herein, we cannot provide a clear‐cut explanation for this observation, since, to our knowledge, no data are available on a direct crosstalk (e.g., heterodimerization) between D2R and SSTR3 on cell membrane (nor in pituitary tumors neither in other lesions)^41^ and data on the modulation of SSTR3 intracellular machinery following D2R targeting are not reported in the literature.

Overall, available evidences suggest that other factors beyond receptor expression, which remain incompletely understood (e.g., expression and modulation of multiple molecules involved in receptor trafficking and intracellular machinery), play a key role in determining the response to medical treatment in NF‐PitNETs.^10^ In particular, multiple intracellular proteins are known to be involved in receptor signal transduction, as well as internalization and recycling (e.g., β‐arrestins, filamin A, cofilin).^10^ Recently, the protein KBTBD6/7 has been shown to play a role in D2R ubiquitination and an inverse correlation between KBTBD6/7 and D2R expression has been observed in multiple PitNET histotypes.^42^ However, due to the intermediate‐high expression of D2R observed in our samples and the superimposable D2R expression in responders vs. non‐responders, we can speculate that KBTBD6/7 does not represent the main mechanism behind the lack of efficacy of DA in our cohort. Similarly, a recent study carried out in PRL‐secreting PitNETs evaluated the possible role of cholesterol in modulating D2R localization, reducing D2R expression on cell membrane, and therefore, the amount of targetable receptor.^43^ However, this mechanism has not been studied in NF‐PitNETs. Other factors, including the splicing machinery, epigenetic regulation of receptor expression or miRNA expression have been involved in determining treatment response in PitNETs.^10^

Focusing on the “responder group,” we observed a strong negative correlation between SSTR1 expression and the response to OCT and PAS alone. SSTR1 expression has been associated with higher aggressiveness in some tumor types (e.g., prostatic cancer, colon cancer),^44^, ^45^ whereas in bronchial NET SSTR1 expression has been associated with improved survival.^46^ In the light of the relatively low number of “responder tumors” analyzed, this observation cannot be generalized.

Interestingly, in the “responder group,” we observed a trend towards better response to medical treatment in invasive tumors compared to the non‐invasive ones. Invasive tumors have a higher recurrence risk due to the higher likelihood of residual tissue after surgery and would therefore benefit most from medical treatment.^15^, ^47^ However, this finding was not observed in the whole cohort, and no difference in receptor expression was observed between invasive and non‐invasive tumors. Moreover, in the “responder group” we observed, for the first time in NF‐PitNETs, a negative correlation between Ki‐67% and the response to the dopaminergic and the chimeric compound. Of note, a similar association has recently been proposed in GH‐secreting PitNETs.^48^ However, due to the low sample size of the responder group, these data need to be confirmed by further studies.

Finally, to the best of our knowledge, this is the first study in which the in vitro response to different medical treatments in NF‐PitNETs has been correlated with the clinic‐pathological classifications of Trouillas and with the recent PANOMEN‐3 classification. Of note, none of the abovementioned classifications was able to identify tumor responders vs. non‐responders to the various tested compounds.

Our study presents some peculiar strengths, as well as some limitations that need to be addressed. One strength is the large cohort of NF‐PitNETs analyzed, in which multiple compounds have been tested simultaneously, which allowed us to perform, despite the limited sample size, a subgroup analysis in the “responder” vs. the “non‐responder group”. Furthermore, multiple potential predictors of response to SRLs and DA treatment have been analyzed, including general patients' characteristics, SSTR/D2R expression, proliferation indices, tumor invasiveness, as well as (for the first time) the Trouillas and the PANOMEN‐3 classifications. On the other hand, one main limitation is the absence of the lineage characterization (e.g., PIT‐1, T‐PIT expression) at histopathological evaluation. Our cohort was composed mainly by tumors classified as gonadotropinomas by hormone IHC, two null cell PitNETs and three silent corticotroph PitNETs. The evaluation of transcription factors could have help to better stratify the tumors, potentially identifying factors able to determine the response to medical treatment. Different PitNET histotypes identified based on transcription factors may have differential SSTR expression, with a potential impact on treatment outcome.^49^

In conclusion, we confirm the limited efficacy of SRL and DA treatment in NF‐PitNETs while highlighting that a subgroup of tumors could benefit from medical treatment (particularly DA agonist). SSTR2 and D2R expression identified tumors responsive only to the chimeric compound BIM‐23B065 (with moderate‐to‐low sensitivity), whereas no other factors reliably predicted responders to the other compounds or identified the tumors belonging to the “responder group.”

Therefore, the classical predictors of response to SSTR and D2R targeted therapies are not useful in NF‐PitNETs. Likewise, the more comprehensive classifications of Trouillas and PANOMEN‐3 did not help. We believe that only a deeper understanding of the complex pathophysiology underlying the heterogeneous group of NF‐PitNETs, performed using as a first approach different methods (e.g., multiomic analyses), could lead in the future to properly identify that 20–30% of tumors that could benefit from SRLs and/or DA treatment.

## AUTHOR CONTRIBUTIONS

**Paolo Nozza:** Resources; investigation; writing – review and editing. **Gianluigi Zona:** Resources; writing – review and editing. **Diego Criminelli Rossi:** Resources; writing – review and editing. **Mara Boschetti:** Resources; writing – review and editing. **Diego Ferone:** Funding acquisition; writing – review and editing; supervision. **Anna Arecco:** Resources; investigation; writing – review and editing. **Jessica Amarù:** Formal analysis; writing – original draft; writing – review and editing; investigation; validation; visualization. **Claudia Campana:** Formal analysis; writing – original draft; writing – review and editing; investigation; validation; visualization. **Federico Gatto:** Conceptualization; formal analysis; supervision; resources; writing – review and editing; project administration; writing – original draft; funding acquisition; visualization. **Marica Arvigo:** Investigation; validation; supervision; conceptualization; writing – review and editing; project administration.

## FUNDING INFORMATION

Open access publishing facilitated by Universita degli Studi di Genova, as part of the Wiley‐CRUI‐CARE agreement. WOA Institution: Universita degli Studi di Genova, Consortia Name: CRUI‐CARE 2024.

## CONFLICT OF INTEREST STATEMENT

C.C. has received advisory board fees from Recordati Rare Diseases. D.F. has received lecture, advisory board, and steering committee fees as well as research grants from Recordati Rare Diseases, Camurus, Novartis‐Advanced Accelerator Applications, Ipsen, and Bristol‐Myers Squibb. F.G. has received lecture/manuscript writing and advisory board fees from Recordati Rare Diseases, Camurus, Ipsen, and Pfizer. The other authors declare no competing interests.

## Supporting information


**Figure S1.** Heatmap plot represents the correlation matrix between in vitro effect of all tested compound (10 nM), alone or in combination, on cell proliferation in 44 primary cultures from NF‐PitNETs. The intensity of the color indicates the strength of the correlation [OCT, octreotide; PAS, pasireotide; BIM‐53097, D2R agonist; BIM‐23B065, SSTR2/SSTR5/D2R chimeric compound].

**Figure S2.** Receiver Operating Characteristic (ROC) curves and the related areas under the curves (AUC) show the ability of receptor expression to identify responders to the different compounds [D2R, dopamine receptor type 2; SSTR2, somatostatin receptor subtype 2; IRS, immunoreactivity score; OCT, octreotide; PAS, pasireotide; BIM‐53097, D2R agonist].

**Table S1.** Univariable linear regression model. Linear regression analysis was computed for the statistically significant correlations observed between treatment efficacy and the continuous variables.

**Table S2.** Tumor response to the different tested conditions. The symbol “X” highlights the positive response (≥20% cell proliferation inhibition) to a specific treatment in the different primary cultures.

## Data Availability

The data that support the findings of this study are available from the corresponding author upon reasonable request.
