# Supplementary material for: Predictors of in vitro response to somatostatin and dopamine receptor‐targeted treatments are lacking in clinically non‐functioning pituitary tumors
Source: J Neuroendocrinol. 2026 Sep 24;38(10):e70272. doi: 10.1111/jne.70272 (PMC13612157; doi:10.1111/jne.70272)
Supplement: Supplementary file 1 [file JNE-38-e70272-s002.pdf]

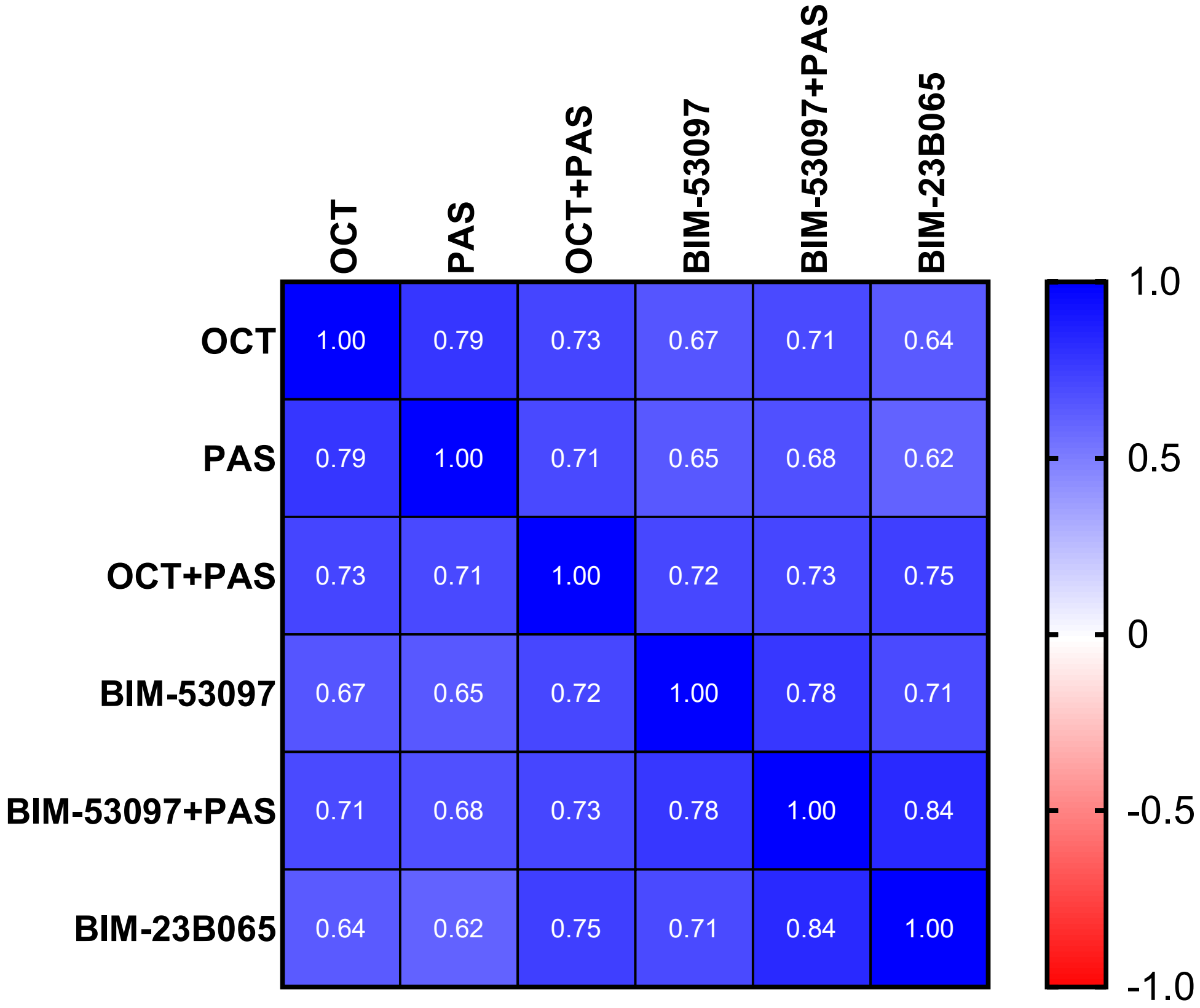

OCT
PAS
OCT+PAS
BIM-53097
BIM-53097+PAS
BIM-23B065
OCT 1.00 0.79 0.73 0.67 0.71 0.64
PAS 0.79 1.00 0.71 0.65 0.68 0.62
OCT+PAS 0.73 0.71 1.00 0.72 0.73 0.75
BIM-53097 0.67 0.65 0.72 1.00 0.78 0.71
BIM-53097+PAS 0.71 0.68 0.73 0.78 1.00 0.84
BIM-23B065 0.64 0.62 0.75 0.71 0.84 1.00
1.0
0.5
0
-0.5
-1.0
