# Supplementary material for: Predictors of in vitro response to somatostatin and dopamine receptor‐targeted treatments are lacking in clinically non‐functioning pituitary tumors
Source: J Neuroendocrinol. 2026 Sep 24;38(10):e70272. doi: 10.1111/jne.70272 (PMC13612157; doi:10.1111/jne.70272)
Supplement: Supplementary file 2 [file JNE-38-e70272-s004.pdf]

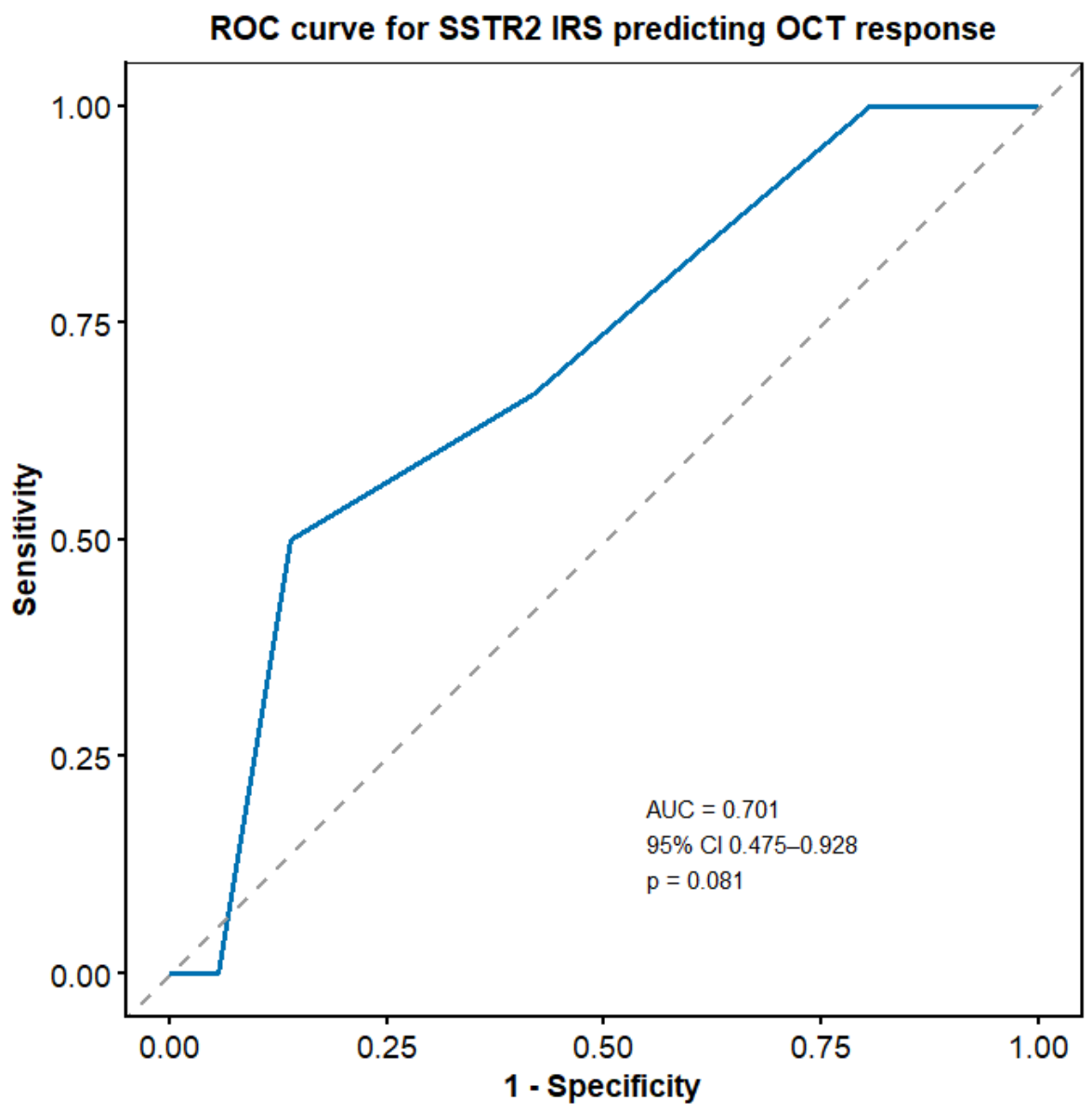
ROC curve for SSTR2 IRS predicting OCT response
Sensitivity
1 - Specificity
0.00
0.25
0.50
0.75
1.00
AUC = 0.701
95% CI 0.475–0.928
p = 0.081

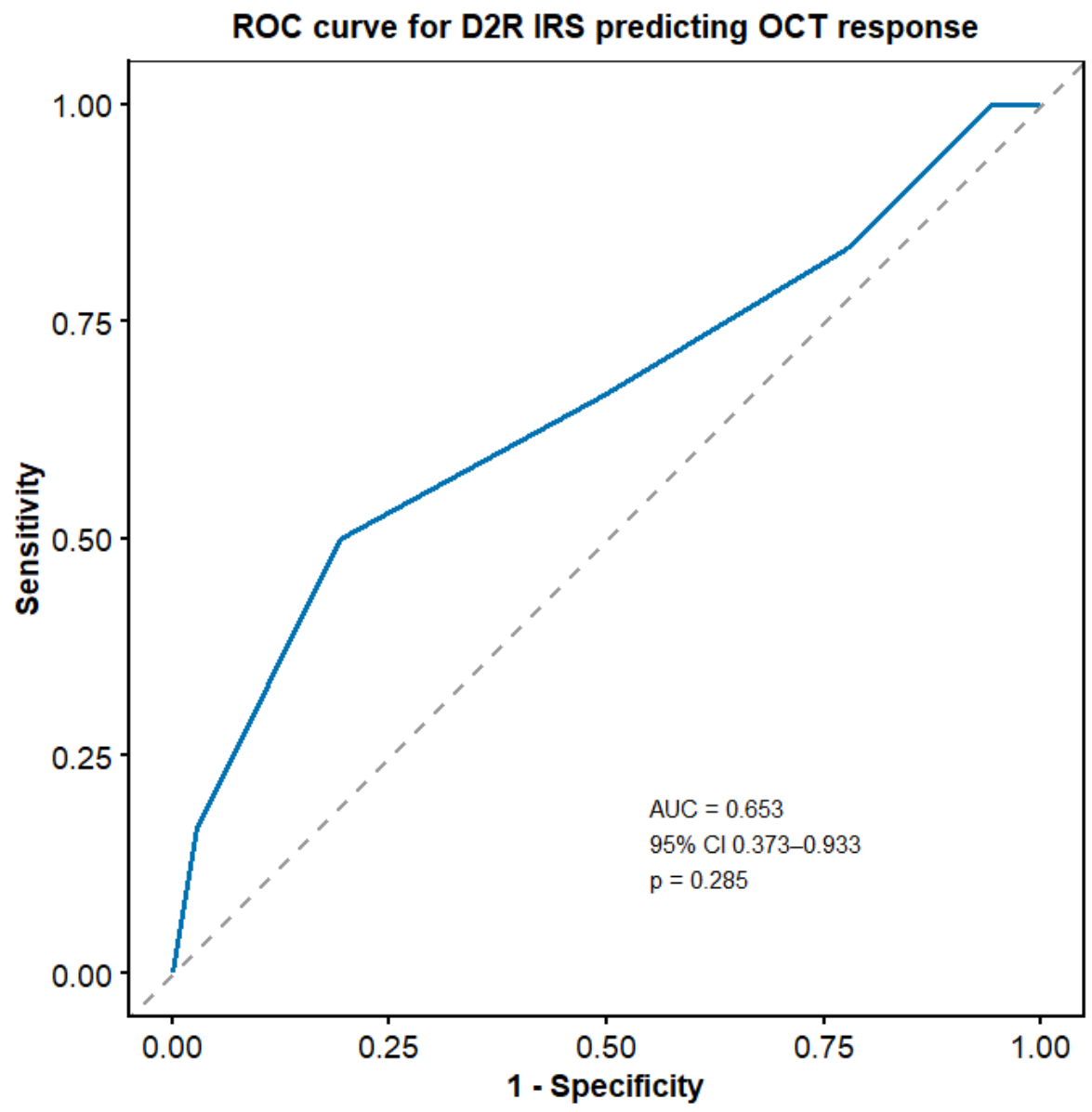
ROC curve for D2R IRS predicting OCT response
Sensitivity
1 - Specificity
0.00
0.25
0.50
0.75
1.00
AUC = 0.653
95% CI 0.373–0.933
p = 0.285

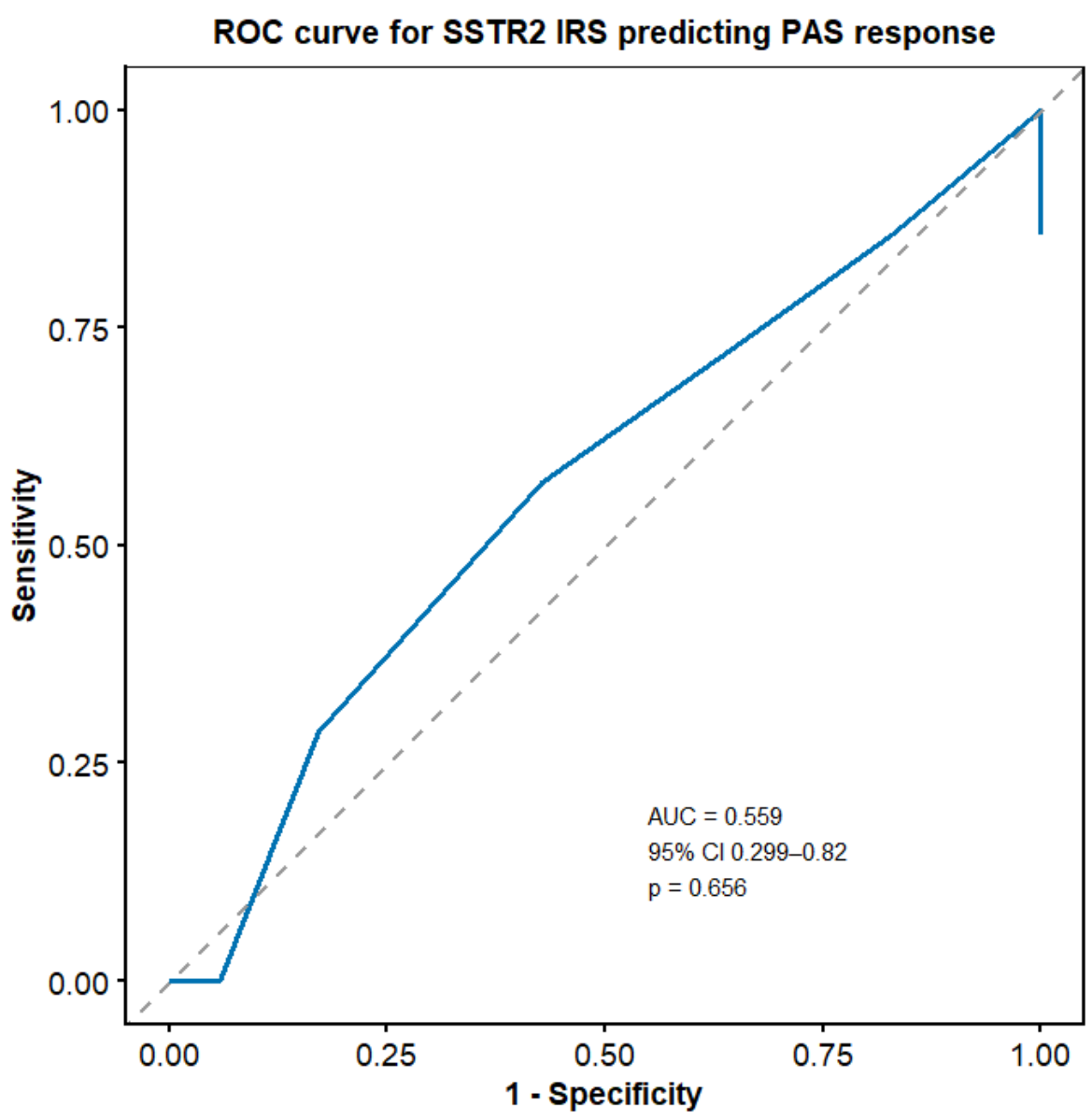
ROC curve for SSTR2 IRS predicting PAS response
Sensitivity
1 - Specificity
0.00
0.25
0.50
0.75
1.00
AUC = 0.559
95% CI 0.299–0.82
p = 0.656

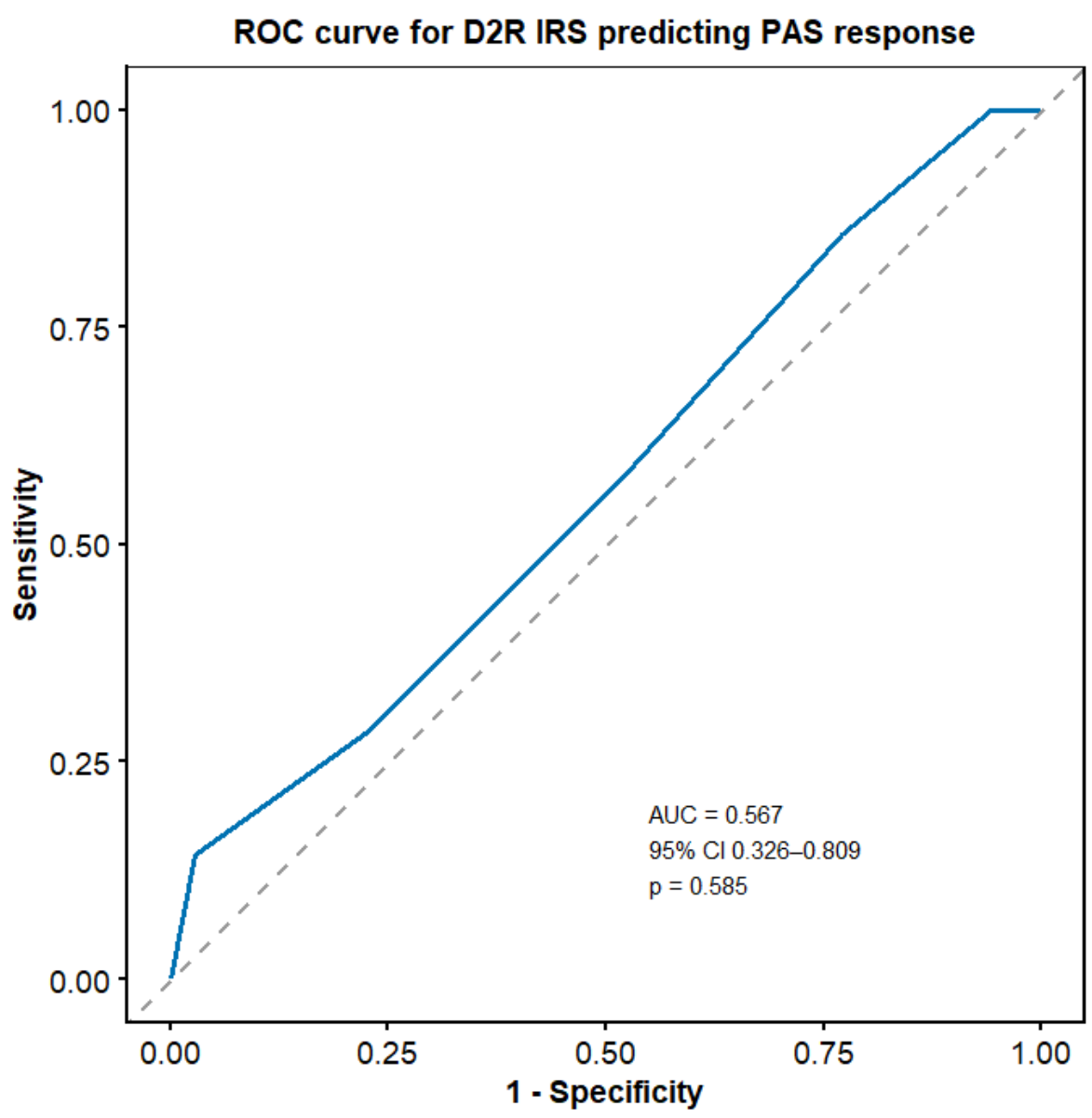
ROC curve for D2R IRS predicting PAS response
Sensitivity
1 - Specificity
0.00
0.25
0.50
0.75
1.00
AUC = 0.567
95% CI 0.326–0.809
p = 0.585

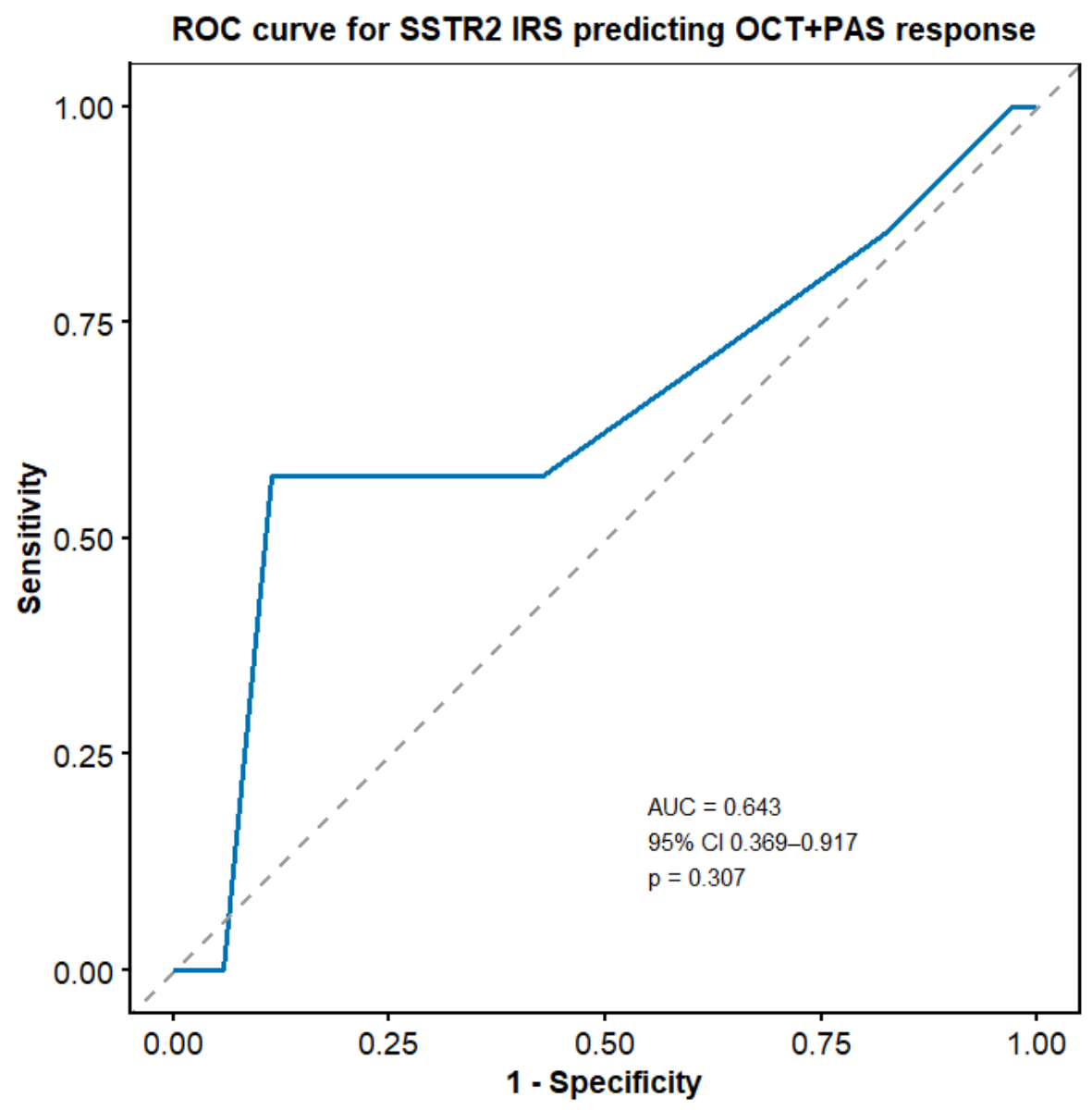
ROC curve for SSTR2 IRS predicting OCT+PAS response
Sensitivity
1 - Specificity
AUC = 0.643
95% CI 0.369–0.917
p = 0.307

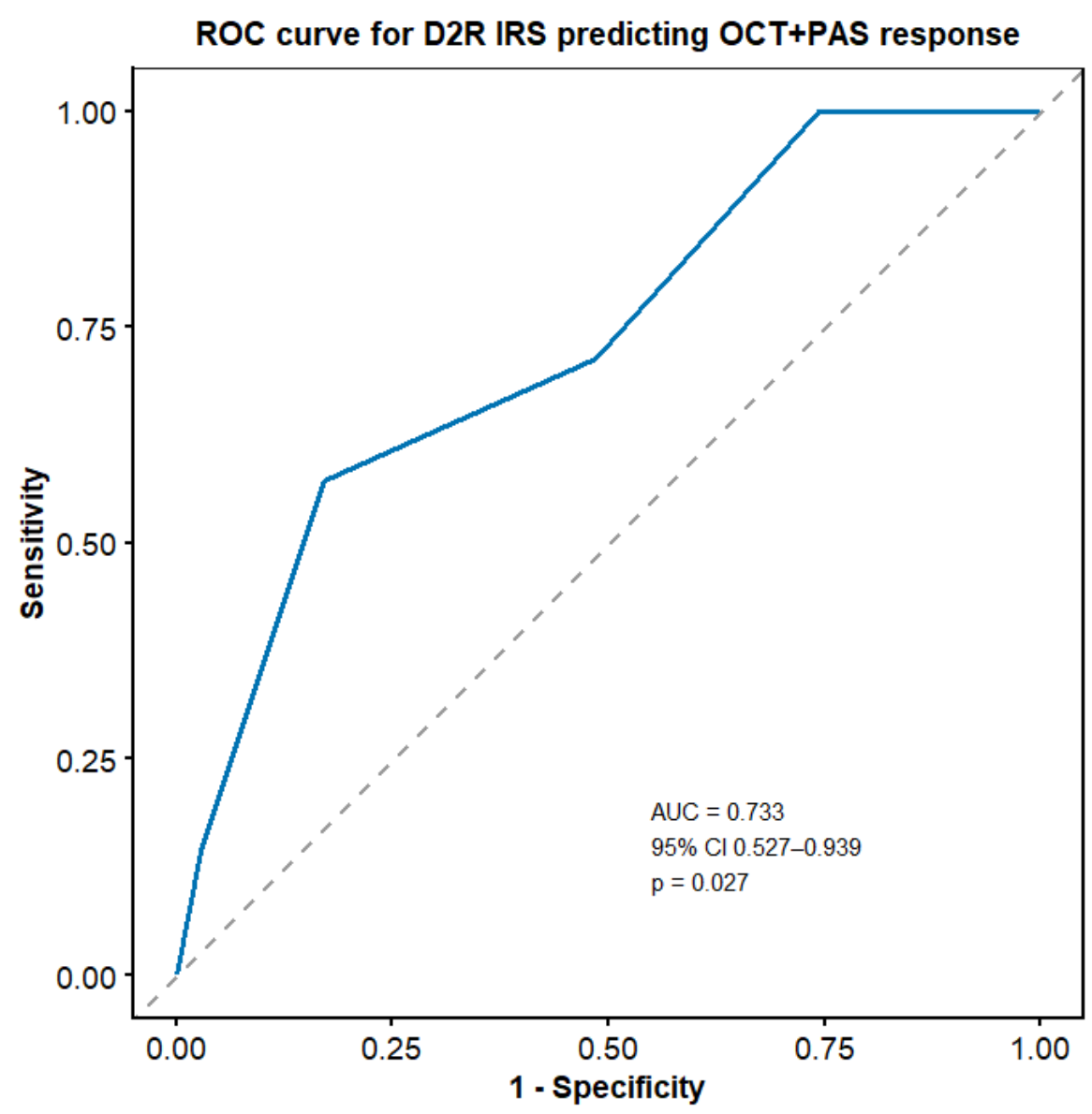
ROC curve for D2R IRS predicting OCT+PAS response
Sensitivity
1 - Specificity
AUC = 0.733
95% CI 0.527–0.939
p = 0.027

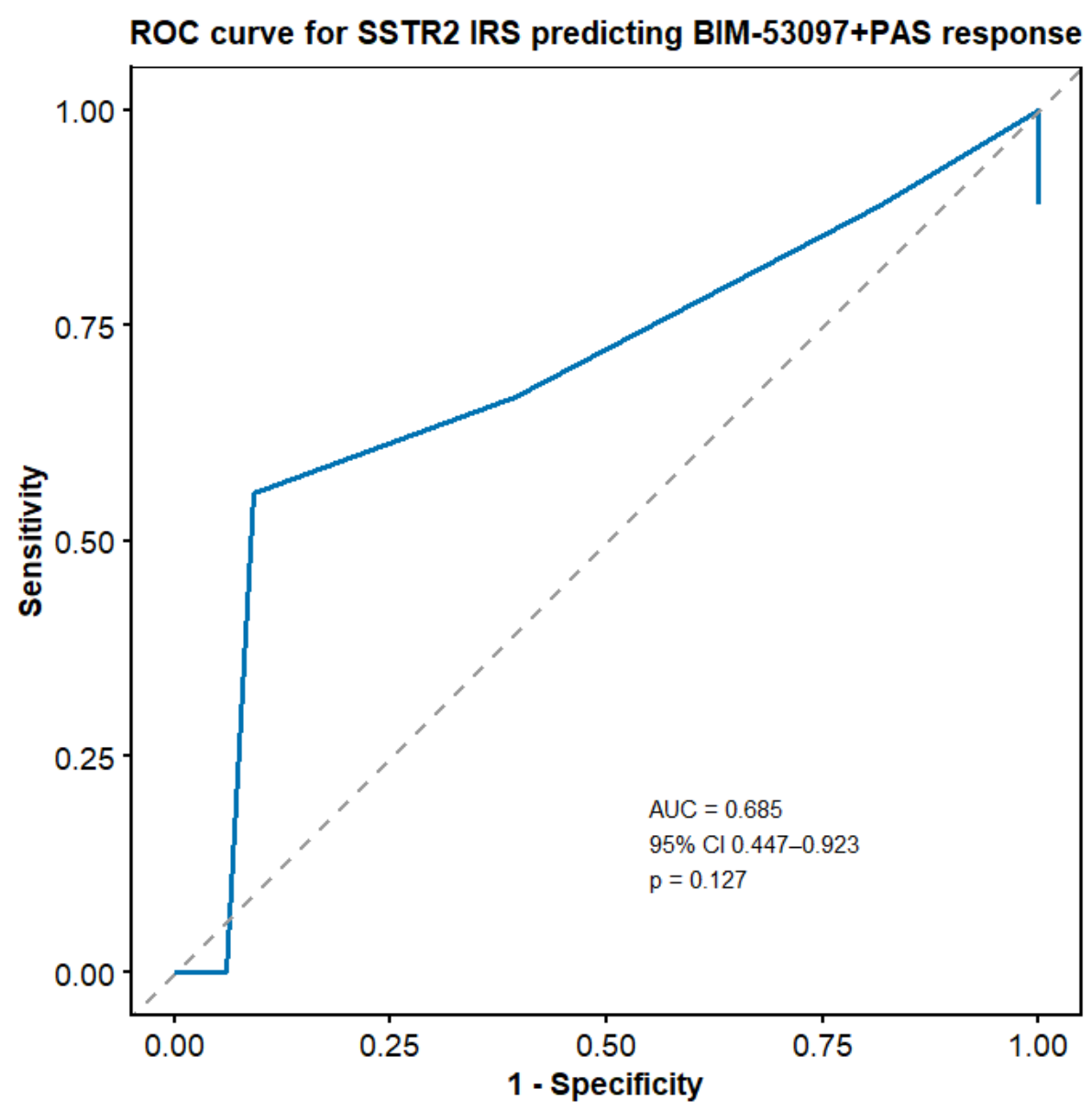
ROC curve for SSTR2 IRS predicting BIM-53097+PAS response
Sensitivity
1 - Specificity
AUC = 0.685
95% CI 0.447–0.923
p = 0.127

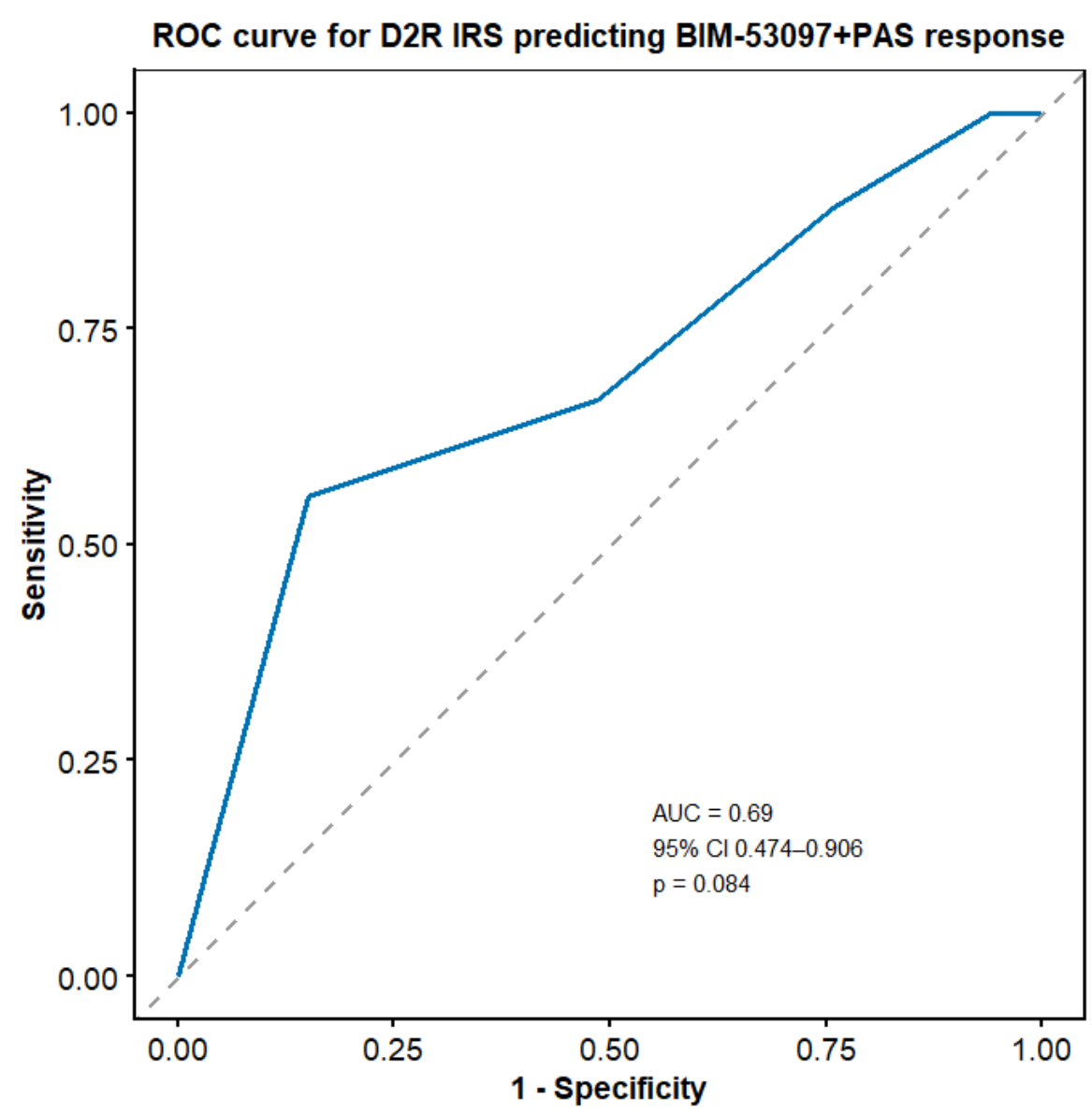
ROC curve for D2R IRS predicting BIM-53097+PAS response
Sensitivity
1 - Specificity
AUC = 0.69
95% CI 0.474–0.906
p = 0.084
