# Supplementary material for: Predictors of in vitro response to somatostatin and dopamine receptor‐targeted treatments are lacking in clinically non‐functioning pituitary tumors
Source: J Neuroendocrinol. 2026 Sep 24;38(10):e70272. doi: 10.1111/jne.70272 (PMC13612157; doi:10.1111/jne.70272)
Supplement: Supplementary file 3 [file JNE-38-e70272-s001.docx]

| Treatment | Variable | β (95% CI) | SE | P value | R^2^ |
| --- | --- | --- | --- | --- | --- |
| OCT | SSTR2 | 2.01 (0.23 - 3.79) | 0.88 | 0.028 | 0.115 |
| PAS | SSTR2 | 1.61 (-0.15 - 3.38) | 0.87 | ns | 0.078 |
|  | Age at surgery | -0.35 (-0.67 - -0.03) | 0.16 | 0.033 | 0.104 |
| OCT+PAS | Age at surgery | -0.46 (-0.81 - -0.11) | 0.17 | 0.011 | 0.145 |
| BIM097 | SSTR3 | -3.39 (-7.10 - 0.33) | 1.84 | ns | 0.078 |
|  | Age at surgery | -0.37 (-0.68 - -0.06) | 0.15 | 0.020 | 0.123 |
| BIM097+PAS | SSTR2 | 2.86 (0.56 - 5.17) | 1.14 | 0.016 | 0.136 |
|  | Age at surgery | -0.47 (-0.89 - -0.04) | 0.21 | 0.032 | 0.105 |

**Supplementary Table 1.** Univariable linear regression model. Linear regression analysis was computed for the statistically significant correlations observed between treatment efficacy and the continuous variables evaluated.
