# Supplementary material for: Predictors of in vitro response to somatostatin and dopamine receptor‐targeted treatments are lacking in clinically non‐functioning pituitary tumors
Source: J Neuroendocrinol. 2026 Sep 24;38(10):e70272. doi: 10.1111/jne.70272 (PMC13612157; doi:10.1111/jne.70272)
Supplement: Supplementary file 4 [file JNE-38-e70272-s003.docx]

**Supplementary Table 2.** Tumor response to the different tested conditions. The symbol “X” highlights the positive response (≥20% cell proliferation inhibition) to a specific treatment in the different primary cultures.

| Tumor  primary  culture (n.) | Patient  (n.) | OCT | PAS | OCT+PAS | BIM-53097 | BIM-53097  +PAS | BIM-23B065 | |
| --- | --- | --- | --- | --- | --- | --- | --- | --- |
| 1 | 1 | **X** |  | **X** | **X** | **X** | | **X** |
| 2 | 2 | **X** | **X** | **X** | **X** | **X** | | **X** |
| 3 | 3 |  | **X** |  | **X** |  | |  |
| 4 | 4 | **X** | **X** | **X** | **X** | **X** | | **X** |
| 5 | 5 |  |  |  |  | **X** | | **X** |
| 6 | 6 |  |  | **X** | **X** | **X** | | **X** |
| 7 | 7 |  |  |  | **X** | **X** | | **X** |
| 8 | 8 |  |  |  |  |  | |  |
| 9 | 9 |  |  |  |  |  | |  |
| 10 | 10 |  |  |  |  |  | |  |
| 11 | 11 | **X** | **X** | **X** | **X** | **X** | | **X** |
| 12 | 12 |  |  |  |  |  | |  |
| 13 | 13 |  |  |  |  |  | |  |
| 14 | 14 |  |  |  |  |  | |  |
| 15 | 15 |  |  |  |  |  | |  |
| 16 | 16 |  |  |  |  |  | |  |
| 17 | 17 |  |  |  |  |  | |  |
| 18 | 18 |  |  |  |  |  | |  |
| 19 | 19 |  |  |  |  |  | |  |
| 20 | 20 |  |  |  |  |  | |  |
| 21 | 5* |  |  | **X** |  |  | |  |
| 22 | 21 |  |  |  |  |  | |  |
| 23 | 22 |  |  |  |  |  | |  |
| 24 | 23 |  |  |  |  |  | |  |
| 25 | 24 |  |  |  |  |  | |  |
| 26 | 25 |  |  |  |  |  | |  |
| 27 | 26 |  | **X** |  | **X** | **X** | |  |
| 28 | 3* |  |  |  |  |  | |  |
| 29 | 27 |  |  |  |  |  | |  |
| 30 | 28 |  |  |  |  |  | |  |
| 31 | 29 |  |  |  |  |  | |  |
| 32 | 30 | **X** | **X** |  |  |  | |  |
| 33 | 31 |  |  |  |  |  | |  |
| 34 | 32 |  |  |  |  |  | |  |
| 35 | 33 |  |  |  |  |  | |  |
| 36 | 34 |  |  |  |  |  | |  |
| 37 | 35 |  |  |  |  |  | |  |
| 38 | 36 |  |  |  |  |  | |  |
| 39 | 37 |  |  |  |  |  | |  |
| 40 | 38 | **X** | **X** | **X** | **X** | **X** | |  |
| 41 | 39 |  |  |  |  |  | |  |
| 42 | 40 |  |  |  |  |  | |  |
| 43 | 41 |  |  |  |  | **X** | | **X** |
| 44 | 20* |  |  |  |  |  | |  |
